# A study on the distribution of high-risk pregnancies and the influencing factors of pregnancy outcomes in Changde City, Hunan Province, China

**DOI:** 10.3389/frph.2026.1775712

**Published:** 2026-05-08

**Authors:** Xiaoying Zhong, Zhaoping Wang, Sisi Hu, Cong Lu, Yao Huang, Jing Zhang, Xuan Ding

**Affiliations:** 1 Department of Obstetrics, The Maternal and Child Health Hospital of Changde City, Changde, Hunan, China; 2 Department of Obstetrics and Gynecology, Changde Hospital, Xiangya School of Medicine, Central South University (The First People’s Hospital of Changde City), Changde, Hunan, China

**Keywords:** distribution, high-risk pregnancy, influencing factors, pregnancy outcome, XGBoost

## Abstract

**Objective:**

To analyze the distribution of risk severity and the influencing factors of pregnancy outcomes among high-risk pregnant women in Changde City, Hunan Province, China.

**Methods:**

A total of 19,301 pregnant women from the jurisdiction of Changde City over the past year (July 2022 to July 2023) were selected as observational subjects. Their demographic information (including age, education level, occupation type, place of permanent residence) and prenatal care data [including gestational week at first check-up, number of prenatal visits, gravida, parity, high-risk assessment (dynamic evaluation), gestational week at delivery, mode of delivery, and maternal and neonatal outcomes] were analyzed and summarized. Univariate and multivariate logistic regression analyses were used to screen variables influencing pregnancy outcomes in high-risk pregnant women. The XGBoost algorithm was employed to construct a model for predicting pregnancy outcomes.

**Results:**

14,196 (73.55%) were initially assessed as high-risk pregnancies. Furthermore, 567 high-risk pregnant women subsequently experienced an escalation in their risk level. Among the 14,196 high-risk pregnant women, the incidences of severe preeclampsia and postpartum hemorrhage were relatively high among adverse maternal outcomes, while the incidence of preterm birth and low birth weight infants was higher among adverse fetal and neonatal outcomes. Univariate and multivariate logistic regression analyses revealed that age, permanent residence, having fewer than 5 prenatal visits, BMI ≥28 kg/m^2^ and pregnancy achieved by assisted reproductive technology significantly influenced pregnancy outcomes (*P* < 0.05). The pregnancy outcome prediction model based on the XGBoost algorithm achieved an area under the curve (AUC) of 0.742 (95% CI: 0.727–0.757) in the training set and an AUC of 0.762 (95% CI: 0.744–0.780) in the test set.

**Conclusion:**

The initial assessment rate of high-risk pregnancy among pregnant women in Changde City is high, and numerous factors influence adverse pregnancy outcomes. Clinically, it is essential to enhance precise screening and tiered management of high-risk pregnancies, implement targeted interventions for core risk factors, and strengthen health education and social support to minimize adverse maternal and infant outcomes and reduce the associated socioeconomic burden.

## 1 Introduction

High-risk pregnancy refers to a condition in which certain pathological or causative factors are present during gestation, posing actual or potential harm to the health of both the pregnant individual and the fetus (1). It results from a combination of various factors, including pre-existing chronic conditions in the pregnant person, environmental influences, and pregnancy-related complications. Moreover, the composition of risk factors for high-risk pregnancy may vary depending on geographic setting, economic status, and other regional differences (2). Therefore, analyzing the local distribution of high-risk pregnancies and identifying the main factors contributing to adverse pregnancy outcomes are essential for improving the management of high-risk pregnant individuals. Such efforts can help enhance tiered management and intervention strategies, thereby reducing the incidence of adverse pregnancy outcomes and alleviating the associated economic burden on families and society.

According to a *Systematic Analysis of Global and Regional Causes of Maternal Death from 2009 to 2020*, released by the World Health Organization in 2025, hemorrhage remains the most common cause of maternal death worldwide (27%), followed by obstetric-related conditions (23%), hypertensive disorders (16%), pregnancy-related infections (7%), embolism (7%), and other direct causes (10%) (3). In China, maternal mortality has declined substantially over the past three decades, from 95.2 per 100,000 in 1990 to 13.6 per 100,000 in 2017, with an annualized reduction rate of 7.0% (4). However, the “Healthy China 2030” Planning Outline sets a national target to further reduce the maternal mortality rate to 12.0 per 100,000 by 2030, emphasizing the need for improved maternal and infant safety initiatives, enhanced screening, and early diagnosis and treatment of maternal diseases (5). Despite significant progress, geographical disparities persist, with maternal mortality rates in western regions remaining notably higher than in central and eastern areas (4).

Within this national context, Hunan Province has implemented a five-color risk management system for pregnancy, which classifies pregnant women into five risk levels (Green, Yellow, Orange, Red, and Purple) to facilitate tiered management and targeted interventions. Changde City, a prefecture-level city in Hunan Province, serves as a representative region for studying the implementation of this system. However, to date, no comprehensive study has analyzed the distribution of high-risk pregnancies across different counties in Changde City or investigated the factors influencing pregnancy outcomes under this policy framework. While previous studies have identified general risk factors for adverse pregnancy outcomes (6–9), they often lack region-specific, policy-anchored analyses that can directly inform local healthcare strategies. Therefore, this study aims to fill this gap by examining the distribution of high-risk pregnancies and the determinants of pregnancy outcomes in Changde City, thereby providing evidence-based recommendations for optimizing tiered management and improving maternal and neonatal health in this region.

## 2 Method

### 2.1 Study design

Pregnant individuals in the jurisdiction of Changde City from July 2022 to July 2023 (a stable phase of local maternal health management policies and prenatal care service provision with a sufficiently large and representative sample in Changde) were selected as the observation subjects for this study. The research protocol was approved by the Medical Ethics Committee of Changde Maternal and Child Health Hospital (Approval Nos.: 2022-IRB-004, 2023-IRB-005, 2024-IRB-007).

Inclusion criteria were as follows: (1) Age between 15 and 50 years; (2) Complete individual case data available; (3) Good compliance, able to cooperate and complete the investigation; (4) Informed consent to participate in the study. Exclusion criteria were as follows: (1) Presence of cognitive dysfunction, verbal communication disorders, or neurological disorders; (2) Individuals with non-local household registration residing in the area for less than 3 months; (3) Loss to follow-up.

### 2.2 Data collection and definitions

Demographic information and prenatal care data of the selected pregnant individuals within the city's jurisdiction were collected. This included age, education level, occupation type, and place of permanent residence. Prenatal care data encompassed gestational week at first check-up, number of prenatal visits, pregnancy order (gravida), parity, high-risk assessment (dynamic evaluation), gestational week at delivery, mode of delivery, and maternal and neonatal outcomes.

1. Diagnostic Criteria for High-Risk Pregnancy:

The criteria were established based on the Implementation and Management Plan for Maternal Pregnancy Risk Assessment in Hunan Province, which adopts the five-color risk classification system in accordance with the Notice of the General Office of the National Health and Family Planning Commission on Issuing the Standards for Pregnancy Risk Assessment and Management for Pregnant and Postpartum Women [Document No. 35 (2017) from the National Health Office's Maternal and Child Health Department]. Risks are categorized and labeled by severity using five colors: Green (low risk), Yellow (general risk), Orange (elevated risk), Red (high risk), and Purple (infectious disease). The definitions are as follows:

(1) **Green:** Indicates low pregnancy risk, with the pregnant individual in generally good health and no identified pregnancy comorbidities or complications.
(2) **Yellow:** Indicates general pregnancy risk, where the pregnant individual has certain underlying risk factors, or stable and mild pregnancy-related comorbidities or complications.
(3) **Orange:** Indicates elevated pregnancy risk. This applies to individuals with a BMI ≥ 28 kg/m^2^ or age ≥ 40 years, or those with severe pregnancy-related comorbidities/complications posing a potential threat to maternal and fetal safety.
(4) **Red:** Indicates high pregnancy risk, where the pregnant individual has serious pregnancy-related comorbidities/complications, and continuing the pregnancy may threaten the pregnant individual's life.
(5) **Purple:** Indicates the pregnant individual has an infectious disease. This purple label may coexist with other color-coded risk labels.

Pregnant individuals identified solely with the Purple label (primarily involving sensitive data such as HIV or syphilis infection) were excluded from this study to protect group privacy. Therefore, the study population included individuals classified as Green, Yellow, Orange, or Red. It is important to note that risk factors related to BMI are categorized into two distinct levels according to the five-color management system: BMI >25 or <18.5 kg/m^2^ is classified as Yellow, while BMI ≥28 kg/m^2^ is classified as Orange. Although these categories are not mutually exclusive, they represent different clinical intervention thresholds. Therefore, we included both as separate variables in our univariate analysis to evaluate their respective impacts on pregnancy outcomes.

2. High-Risk Pregnancy Outcomes (6):
  (1) **Adverse Maternal Outcomes Included:** Hypertensive heart disease in pregnancy, eclampsia, severe preeclampsia, thyroid storm, diabetic ketoacidosis, severe infection, uterine rupture, postpartum hemorrhage (blood loss ≥500 mL for vaginal delivery or ≥1,000 mL for cesarean section within 24 h of delivery), peripartum hysterectomy, occurrence of severe maternal morbidity (according to WHO criteria), and maternal mortality.
  (2) **Adverse Fetal and Neonatal Outcomes Included:** Spontaneous abortion, medically indicated abortion/induction, stillbirth, fetal death, preterm birth (delivery before 37 weeks of gestation), low birth weight infant (<2,500 g), macrosomia, severe neonatal asphyxia, and congenital anomalies. Due to the time frame of this study, long-term neonatal outcomes such as cerebral palsy were excluded but warrant follow-up investigation in subsequent research.

### 2.3 Quality control

A three-tier quality inspection system encompassing medical institutions, district/county maternal and child health hospitals, and the municipal maternal and child health hospital was established in accordance with the *China Maternal and Child Health Monitoring Program*, with quality control implemented at each level. District and county maternal and child health hospitals collected information on newly registered pregnant individuals from medical institutions across all levels within their jurisdiction each month for archival management. They supervised primary-level doctors and nurses, who had undergone training, assessment, and held valid “Maternal and Child Healthcare Technical Qualification Certificates,” to promptly enter the verified information of pregnant individuals into the *Hunan Provincial Primary Health Information System—Public Health 3.0, thereby creating electronic health records. Concurrently, quality control was performed on these electronic records to verify data authenticity, check for omissions, missing items, or unmanaged cases. The requirements included a 100% archival rate, 100% completeness of record forms, an error rate in form entries of <1%, and a data entry error rate of <0.1%. The municipal maternal and child health hospital conducted monthly online quality control checks by randomly sampling 10 electronic records from each district/county. Identified issues were fed back for rectification. Furthermore, it organized bi-annual on-site specialized training sessions on *Standardized Management of High-Risk Pregnant Women* for district/county staff and performed quality control assessments on their work to ensure standardization and data accuracy. Under the leadership of the Municipal Health Commission, the municipal maternal and child health institution conducted bi-annual reviews of severe maternal cases. These reviews were mandated to cover all aspects of maternal health management within the jurisdiction and participating institutions, aiming to identify systemic gaps and emphasize the timeliness, accuracy, dynamic nature, and personalized guidance required in the management of high-risk pregnant women.

### 2.4 Statistics

Data processing and analysis were performed using R version 4.4.1 and Python 3.12.0. Continuous variables were described using mean ± standard deviation, while categorical variables were summarized using frequencies and constituent percentages. For group comparisons, the chi-square test was employed for categorical variables. For continuous variables, the independent samples t-test was used if the data followed a normal distribution; otherwise, the Mann–Whitney *U*-test was applied. Variables potentially influencing pregnancy outcomes were initially screened using univariate logistic regression. Significant variables were then entered into a multivariate logistic regression model for further selection. Subsequently, the identified variables were incorporated into the XGBoost machine learning algorithm to construct a risk prediction model. As an optimized gradient boosting machine learning method, XGBoost demonstrates strong performance in processing structured clinical data, effectively capturing nonlinear relationships and complex interactions among multiple maternal risk factors, while also exhibiting good resistance to overfitting, high prediction accuracy, and robustness in handling imbalanced outcome data commonly observed in pregnancy-related studies (7). The XGBoost model was constructed using the 5 variables identified by the multivariate logistic regression analysis (*P* < 0.05). The dataset was randomly split into a training set (60%) and a test set (40%). Hyperparameter tuning was performed using a grid search strategy combined with 5-fold cross-validation on the training set. The grid searched over parameters including max_depth (3, 6, 9), learning_rate (0.01, 0.05, 0.1), and n_estimators (100, 200, 300). The parameter set that yielded the highest average AUC across the 5 folds was selected for the final model. The model was further interpreted using SHapley Additive exPlanations (SHAP) analysis. A two-sided *P*-value < 0.05 was considered statistically significant.

## 3 Result

### 3.1 Incidence of high-risk pregnancies and adverse maternal/fetal-neonatal outcomes in Changde City

A total of 19,301 pregnant women within the jurisdiction of Changde City from July 2022 to July 2023 were included in this study. Among them, 14,196 cases with complete data were identified as high-risk pregnancies at the initial examination, resulting in an initial high-risk pregnancy detection rate of 73.55% (14,196/19,301). Furthermore, the risk level escalated during subsequent follow-up for 567 of these initially identified high-risk pregnancies. The pregnancy outcomes for the 14,196 high-risk pregnant women are presented in Table 1. Among adverse maternal outcomes, severe preeclampsia (0.8%) and postpartum hemorrhage (0.3%) were most frequent. For fetal and neonatal outcomes, preterm birth/low birth weight (8.5%) had the highest incidence, followed by macrosomia (5.2%) (Table 1).

**Table 1:** The pregnancy outcomes of 14,196 pregnant women.

Adverse pregnancy outcomes	Group	Case	Percentage
Maternal adverse pregnancy outcomes	Severe preeclampsia	118	0.8%
	Postpartum hemorrhage	38	0.3%
	Diabetic ketoacidosis	1	<0.1%
	Severe infection	1	<0.1%
	Maternal death	1	<0.1%
	Uterine rupture	1	<0.1%
Fetal and neonatal adverse outcomes	Premature infants and low birth weight infants	1,205	8.5%
	Macrocephalic infants	738	5.2%
	Spontaneous abortion	36	0.3%
	Congenital abnormalities	32	0.2%
	Stillbirth	13	<0.1%
	Medical indication-induced induced abortion	9	<0.1%
	Severe neonatal asphyxia	8	<0.1%

### 3.2 Distribution of high-risk pregnancy severity in Changde City

**Figure 1:**
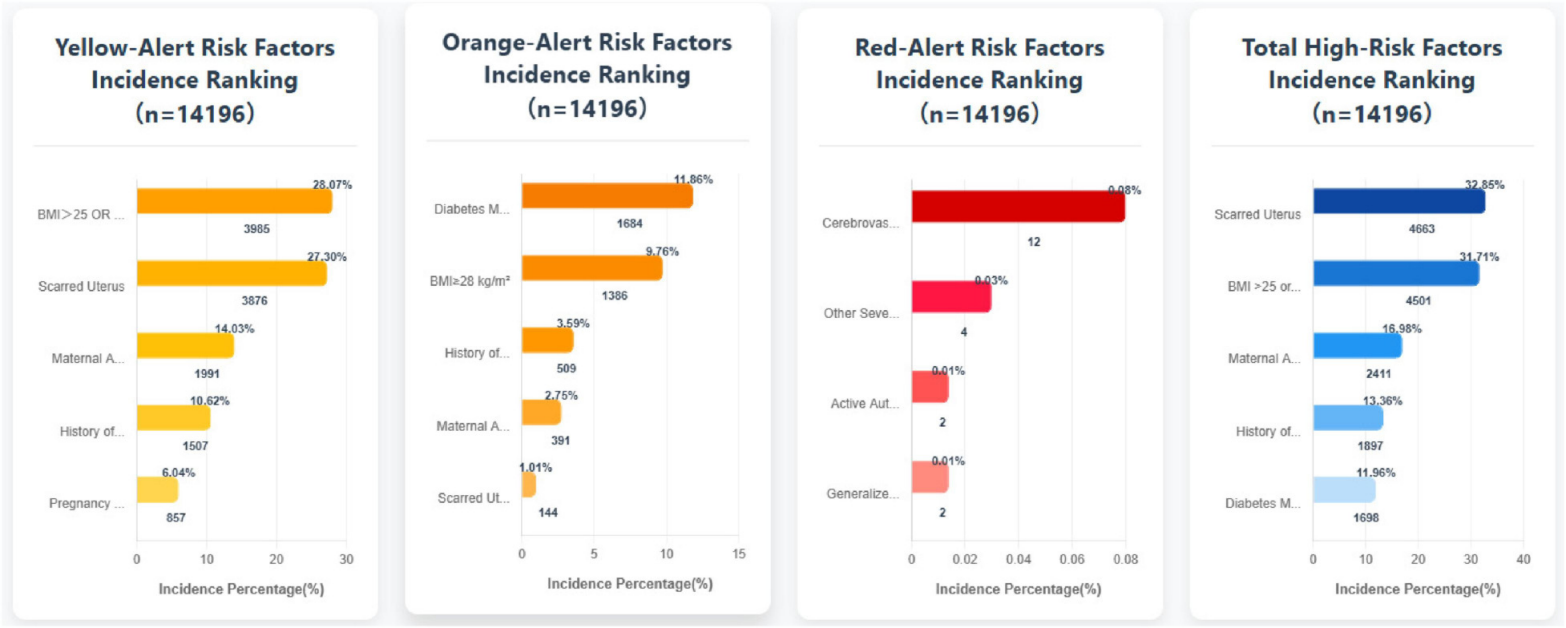
Distribution of the top five high-risk factors in the yellow, orange, red, and overall high-risk pregnancy categories.

Among the 14,196 studied high-risk pregnancy cases, pregnancy risk levels were dynamically adjusted based on prenatal examination results and puerperium conditions. Statistical analysis was conducted according to the highest risk level recorded before case closure. The distribution primarily included Yellow (General Risk), Orange (Elevated Risk), and Red (High Risk) categories. Within the Yellow (General Risk) category, the top five high-risk factors were: BMI >25 or <18.5 kg/m², Scarred Uterus, Maternal Age >35 or ≤18 years, History of Adverse Pregnancy/Obstetric Outcomes, and Pregnancy Complicated with Anemia (Hb 60–110 g/L). Within the Orange (Elevated Risk) category, the top five factors were: Diabetes Mellitus, Thyroid Disorders, or Pituitary Prolactinoma Requiring Medication; BMI ≥28 kg/m²; History of ≥2 Uterine Surgeries; Maternal Age ≥40 years; and Scarred Uterus (with <18 months interval since the last surgery). Within the Red (High Risk) category, the top four factors were: Cerebrovascular Malformation and Surgical History; Other Severe Internal/Surgical Diseases; Active Autoimmune/Immune System Diseases; and Generalized Epileptic Seizures. The overall top five high-risk factors across all categories were: Scarred Uterus, BMI >25 or <18.5 kg/m², Maternal Age >35 or ≤18 years, History of Adverse Pregnancy/Obstetric Outcomes, and Diabetes Mellitus, Thyroid Disorders, or Pituitary Prolactinoma Requiring Medication (Figure 1).

**Figure 2:**
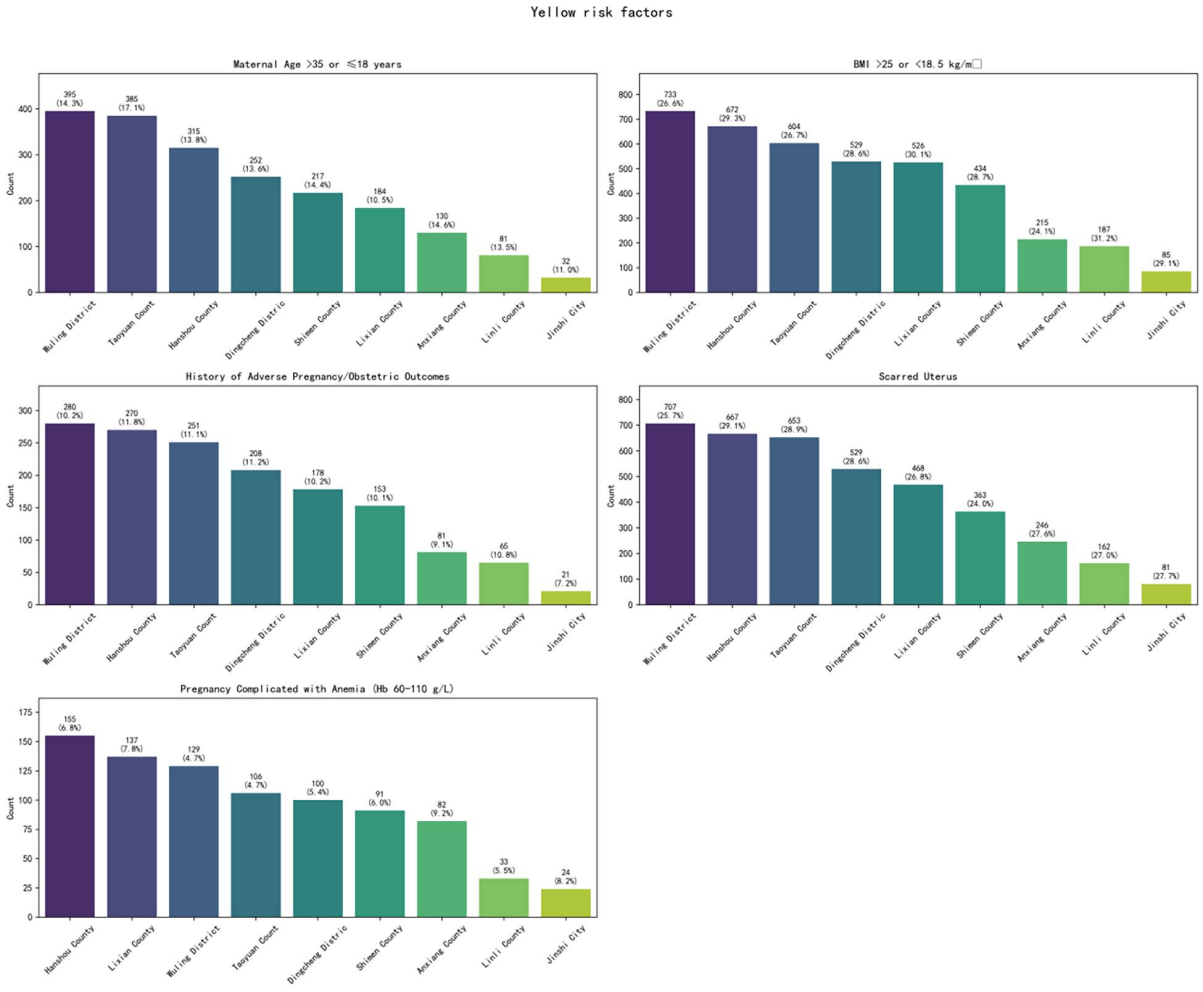
Regional distribution of yellow high-risk factors in Changde City.

**Figure 3:**
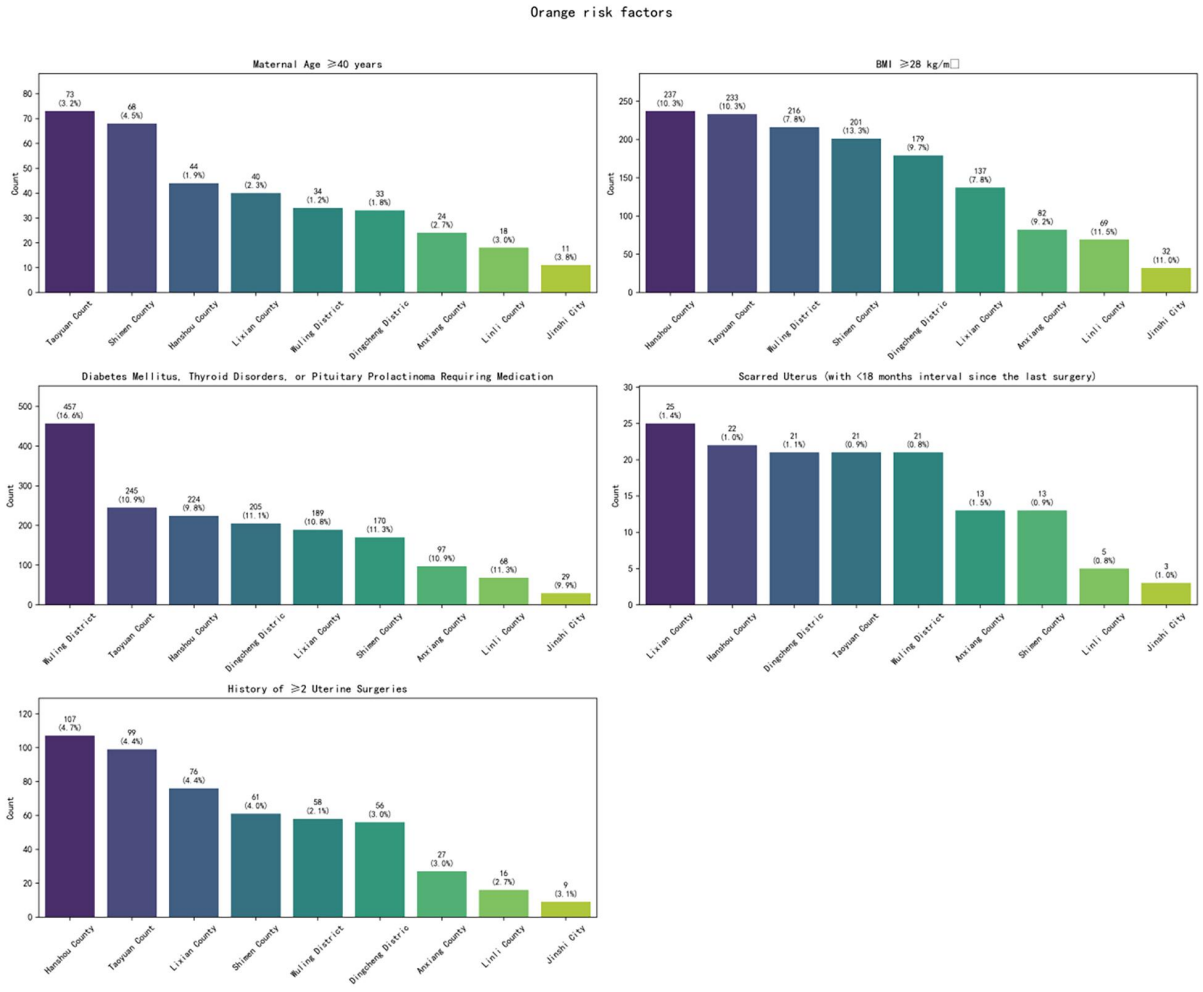
Regional distribution of orange high-risk factors in Changde City.

**Figure 4:**
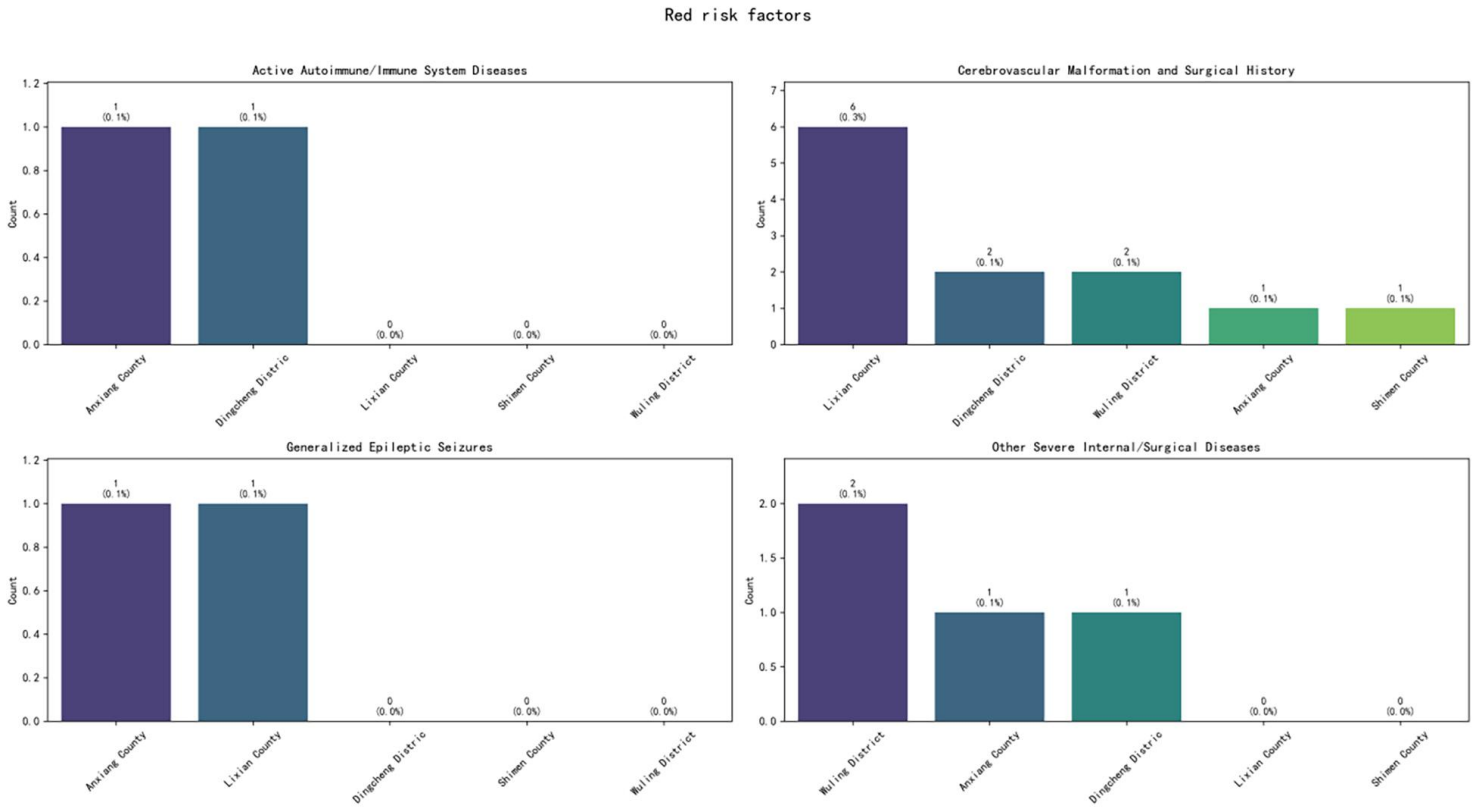
Regional distribution of red high-risk factors in Changde City.

Regarding the county-level distribution of high-risk factors: For Yellow risk factors, Wuling District ranked first in the proportion of discharged cases for four factors: Maternal Age >35 or ≤18 years, BMI >25 or <18.5 kg/m², History of Adverse Pregnancy/Obstetric Outcomes, and Scarred Uterus. It ranked third for Pregnancy Complicated with Anemia (Hb 60–110 g/L). Hanshou County and Taoyuan County also featured prominently in the top proportions for these five factors (Figure 2). For Orange risk factors, Wuling District ranked first in the proportion of discharged cases for Maternal Age ≥40 years and for Diabetes Mellitus, Thyroid Disorders, or Pituitary Prolactinoma Requiring Medication. Hanshou County ranked first for BMI ≥28 kg/m² and for History of ≥2 Uterine Surgeries. Lixian County ranked first for Scarred Uterus (with <18 months interval since the last surgery). Notably, Taoyuan County consistently ranked second for factors including Maternal Age ≥40 years, BMI ≥28 kg/m², Diabetes Mellitus/Thyroid Disorders/Prolactinoma Requiring Medication, and History of ≥2 Uterine Surgeries (Figure 3). For Red risk factors, the overall number of cases was relatively low. However, Lixian County had a higher proportion of discharged cases for Cerebrovascular Malformation and Surgical History, followed by Wuling District and Dingcheng District (Figure 4).

### 3.3 Comparison of general characteristics based on pregnancy outcome grouping

Among the 14,196 high-risk pregnant women, a total of 2,132 experienced adverse maternal outcomes, adverse fetal/neonatal outcomes, or both, resulting in an incidence rate of 15.02%. A comparison of baseline characteristics between the normal pregnancy outcome group and the adverse pregnancy outcome group revealed significant differences in maternal age and gestational week at delivery (*P* < 0.05). The adverse outcome group tended to be older and delivered at an earlier gestational age (Table 2). Furthermore, during the high-risk factor assessment, significant differences (*P* < 0.05) were observed between the two groups for the following indicators: whether the number of prenatal visits was less than 5, presence of a scarred uterus, BMI >25 or <18.5 kg/m^2^, maternal age >35 or ≤18 years, BMI ≥28 kg/m^2^, pregnancy achieved by assisted reproductive technology (ART), whether the risk level was upgraded, presence of multiple high-risk factors, and mode of delivery. Details are provided in Table 2.

**Table 2:** Descriptions of baseline indicator.

Indicator	Total Population (*n* = 14,196)	Normal Pregnancy Outcomes (*n* = 12,064)	Adverse Pregnancy Outcomes (*n* = 2,132)	Statistic	*P*
Age	31.04 ± 4.80	30.94 ± 4.80	31.59 ± 4.75	*t* = −5.74	<0.001
Gestational week at first check-up	11.00 (9.00, 12.00)	11.00 (9.00, 12.00)	11.00 (9.00, 12.00)	*Z* = −1.15	0.248
Gravida	2.00 (2.00, 3.00)	2.00 (2.00, 3.00)	2.00 (1.00, 3.00)	*Z* = −1.57	0.117
Parity	1.00 (0.00, 1.00)	1.00 (0.00, 1.00)	1.00 (0.00, 1.00)	Z = −1.48	0.14
Gestational week at delivery	39.00 (38.00, 40.00)	39.00 (39.00, 40.00)	37.00 (35.00, 39.00)	*Z* = −Inf	<0.001
Education, *n* (%)				*χ*² = 1.73	0.784
Illiterate/Semi-literate & Primary School	430 (3.03)	371 (3.08)	59 (2.77)		
Junior High School	3,907 (27.52)	3,301 (27.36)	606 (28.42)		
Senior High School	5,447 (38.37)	4,644 (38.49)	803 (37.66)		
College/University & Above	4,407 (31.04)	3,744 (31.03)	663 (31.10)		
Unknown	5 (0.04)	4 (0.03)	1 (0.05)		
Occupation, *n* (%)				χ² = 7.92	0.441
Unemployed	1,741 (12.26)	1,492 (12.37)	249 (11.68)		
Other occupations not elsewhere classified	3,937 (27.73)	3,342 (27.70)	595 (27.91)		
Commercial and service workers	1,183 (8.33)	1,000 (8.29)	183 (8.58)		
Clerical and related workers	592 (4.17)	492 (4.08)	100 (4.69)		
Production, transportation equipment operators and related workers	253 (1.78)	219 (1.82)	34 (1.59)		
Heads of state organs, party-mass organizations, enterprises and institutions	163 (1.15)	133 (1.10)	30 (1.41)		
Agricultural, forestry, animal husbandry, fishery, and water conservancy workers	5,094 (35.88)	4,316 (35.78)	778 (36.49)		
Professionals and technical personnel	1,224 (8.62)	1,062 (8.80)	162 (7.60)		
Student	9 (0.06)	8 (0.07)	1 (0.05)		
Residence, *n* (%)				χ² = 13.16	0.107
Wuling District	2,755 (19.41)	2,337 (19.37)	418 (19.61)		
Hanshou County	2,290 (16.13)	1,941 (16.09)	349 (16.37)		
Taoyuan County	2,258 (15.91)	1,965 (16.29)	293 (13.74)		
Dingcheng District	1,852 (13.05)	1,582 (13.11)	270 (12.66)		
Lixian County	1,746 (12.30)	1,485 (12.31)	261 (12.24)		
Shimen County	1,511 (10.64)	1,266 (10.49)	245 (11.49)		
Anxiang County	892 (6.28)	743 (6.16)	149 (6.99)		
Linli County	600 (4.23)	499 (4.14)	101 (4.74)		
Jinshi City	292 (2.06)	246 (2.04)	46 (2.16)		
Number of prenatal visits <5, *n* (%)				χ² = 1,392.82	<0.001
No	13,644 (96.11)	11,902 (98.66)	1,742 (81.71)		
Yes	552 (3.89)	162 (1.34)	390 (18.29)		
Scarred uterus, *n* (%)				χ² = 6.33	0.012
No	9,533 (67.15)	8,051 (66.74)	1,482 (69.51)		
Yes	4,663 (32.85)	4,013 (33.26)	650 (30.49)		
BMI >25 or <18.5 kg/m^2^, *n* (%)				χ² = 5.87	0.015
No	9,695 (68.29)	8,191 (67.90)	1,504 (70.54)		
Yes	4,501 (31.71)	3,873 (32.10)	628 (29.46)		
Maternal age >35 or ≤18 years, *n* (%)				χ² = 9.36	0.002
No	11,785 (83.02)	10,064 (83.42)	1,721 (80.72)		
Yes	2,411 (16.98)	2,000 (16.58)	411 (19.28)		
History of adverse pregnancy, *n* (%)				χ² = 3.50	0.061
No	12,299 (86.64)	10,479 (86.86)	1,820 (85.37)		
Yes	1,897 (13.36)	1,585 (13.14)	312 (14.63)		
Diabetes mellitus, thyroid disorders, or pituitary prolactinoma requiring medication, *n* (%)				χ² = 0.01	0.943
No	12,498 (88.04)	10,622 (88.05)	1,876 (87.99)		
Yes	1,698 (11.96)	1,442 (11.95)	256 (12.01)		
BMI ≥28 kg/m^2^, *n* (%)				χ² = 52.64	<0.001
No	12,809 (90.23)	10,977 (90.99)	1,832 (85.93)		
Yes	1,387 (9.77)	1,087 (9.01)	300 (14.07)		
Pregnancy complicated with anemia, *n* (%)				χ² = 3.34	0.068
No	13,198 (92.97)	11,196 (92.81)	2,002 (93.90)		
Yes	998 (7.03)	868 (7.19)	130 (6.10)		
Pregnancy achieved by ART, *n* (%)				χ² = 102.43	<0.001
No	13,282 (93.56)	11,393 (94.44)	1,889 (88.60)		
Yes	914 (6.44)	671 (5.56)	243 (11.40)		
History of ≥2 uterine surgeries, *n* (%)				χ² = 0.84	0.360
No	13,685 (96.40)	11,637 (96.46)	2,048 (96.06)		
Yes	511 (3.60)	427 (3.54)	84 (3.94)		
History of pelvic surgery, *n* (%)				χ² = 0.44	0.509
No	13,775 (97.03)	11,711 (97.07)	2,064 (96.81)		
Yes	421 (2.97)	353 (2.93)	68 (3.19)		
Risk level upgraded, *n* (%)				χ² = 140.63	<0.001
No	13,629 (96.01)	11,681 (96.83)	1,948 (91.37)		
Yes	567 (3.99)	383 (3.17)	184 (8.63)		
Multiple high-risk factors, *n* (%)				χ² = 306.92	<0.001
No	6,212 (43.76)	5,649 (46.83)	563 (26.41)		
Yes	7,984 (56.24)	6,415 (53.17)	1,569 (73.59)		
Mode of delivery, *n* (%)				χ² = 616.65	<0.001
Vaginal delivery	5,076 (35.76)	4,549 (37.71)	527 (24.72)		
Cesarean section	9,030 (63.61)	7,515 (62.29)	1,515 (71.06)		
Induced labor and abortion	90 (0.63)	0 (0.00)	90 (4.22)		

*t*, t-test; *Z*, Mann–Whitney test, *χ*², Chi-square test.

### 3.4 Univariate and multivariate logistic regression analysis of key factors influencing pregnancy outcomes

Univariate logistic regression analysis identified several factors significantly associated with pregnancy outcomes (*P* < 0.05), including maternal age, gravida (number of pregnancies), gestational week at delivery, permanent residence (specifically Taoyuan County), having fewer than 5 prenatal visits, scarred uterus, BMI >25 or <18.5 kg/m², maternal age >35 or ≤18 years, BMI ≥28 kg/m², pregnancy achieved by ART, whether the risk level was upgraded, presence of multiple high-risk factors, and mode of delivery (Table 3). Then we excluded the causal factors of adverse pregnancy outcomes, including the gestational week at delivery, whether the risk level was upgraded, the presence of multiple high-risk factors, and the mode of delivery. Variables from the univariate analysis with a potential association (*P* < 0.05) were subsequently entered into a multivariate logistic regression model. This refined analysis revealed that age, permanent residence (Anxiang County), having fewer than 5 prenatal visits, BMI ≥28 kg/m² and pregnancy achieved by ART were independently and significantly associated with the risk of adverse pregnancy outcomes (*P* < 0.05) (Table 3).

**Table 3:** Univariate logistic regression analysis and multivariate logistic regression analysis.

Indicators	univariate logistic regression analysis		multivariate stepwise backward logistic regression analysis	
	*P*	OR (95%CI)	*P*	OR (95%CI)
Age	<0.001	1.03 (1.02–1.04)	0.001	1.02 (1.01–1.03)
Gestational week at first check-up	0.717	1.00 (0.98–1.01)		
Gravida	0.021	1.04 (1.01–1.07)	0.163	1.03 (0.99–1.07)
Parity	0.323	0.96 (0.90–1.04)		
Education				
Illiterate/Semi-literate & Primary School		1.00 (Reference)		
Junior High School	0.329	1.15 (0.87–1.54)		
Senior High School	0.565	1.09 (0.82–1.45)		
College/University & Above	0.463	1.11 (0.84–1.48)		
Unknown	0.688	1.57 (0.17–14.31)		
Occupation				
Unemployed		1.00 (Reference)		
Other occupations not elsewhere classified	0.428	1.07 (0.91–1.25)		
Commercial and service workers	0.383	1.10 (0.89–1.35)		
Clerical and related workers	0.127	1.22 (0.95–1.57)		
Production, transportation equipment operators and related workers	0.713	0.93 (0.63–1.37)		
Heads of state organs, party-mass organizations, enterprises and institutions	0.158	1.35 (0.89–2.05)		
Agricultural, forestry, animal husbandry, fishery, and water conservancy workers	0.328	1.08 (0.93–1.26)		
Professionals and technical personnel	0.408	0.91 (0.74–1.13)		
Student	0.786	0.75 (0.09–6.01)		
Residence				
Wuling District		1.00 (Reference)		1.00 (Reference)
Hanshou County	0.947	1.01 (0.86–1.17)	0.865	0.99 (0.84–1.16)
Taoyuan County	0.027	0.83 (0.71–0.98)	0.092	0.86 (0.73–1.02)
Dingcheng District	0.579	0.95 (0.81–1.13)	0.592	1.05 (0.88–1.25)
Lixian County	0.838	0.98 (0.83–1.16)	0.256	1.11 (0.93–1.32)
Shimen County	0.369	1.08 (0.91–1.28)	0.518	0.94 (0.78–1.13)
Anxiang County	0.273	1.12 (0.91–1.38)	0.022	1.28 (1.04–1.59)
Linli County	0.308	1.13 (0.89–1.44)	0.738	0.96 (0.74–1.24)
Jinshi City	0.793	1.05 (0.75–1.46)	0.454	0.87 (0.60–1.25)
Number of prenatal visits <5	<0.001	16.45 (13.60–19.89)	<0.001	16.39 (13.51–19.89)
Scarred uterus	0.012	0.88 (0.80–0.97)	0.244	0.94 (0.84–1.05)
BMI>25 or <18.5 kg/m^2^	0.015	0.88 (0.80–0.98)	0.181	1.08 (0.96–1.21)
Maternal age >35 or ≤18 years	0.002	1.20 (1.07–1.35)	0.185	1.10 (0.95–1.27)
History of adverse pregnancy	0.061	1.13 (0.99–1.29)		
Diabetes mellitus, thyroid disorders, or pituitary prolactinoma requiring medication	0.943	1.01 (0.87–1.16)		
BMI≥28 kg/m^2^	<0.001	1.65 (1.44–1.90)	<0.001	1.80 (1.55–2.10)
Pregnancy complicated with anemia	0.068	0.84 (0.69–1.01)		
Pregnancy achieved by ART	<0.001	2.18 (1.87–2.55)	<0.001	2.06 (1.73–2.44)
History of ≥2 uterine surgeries	0.360	1.12 (0.88–1.42)		
History of pelvic surgery	0.509	1.09 (0.84–1.42)		

OR, Odds Ratio; CI, Confidence Interval.

### 3.5 Establishment of a pregnancy outcome risk prediction model

**Figure 5:**
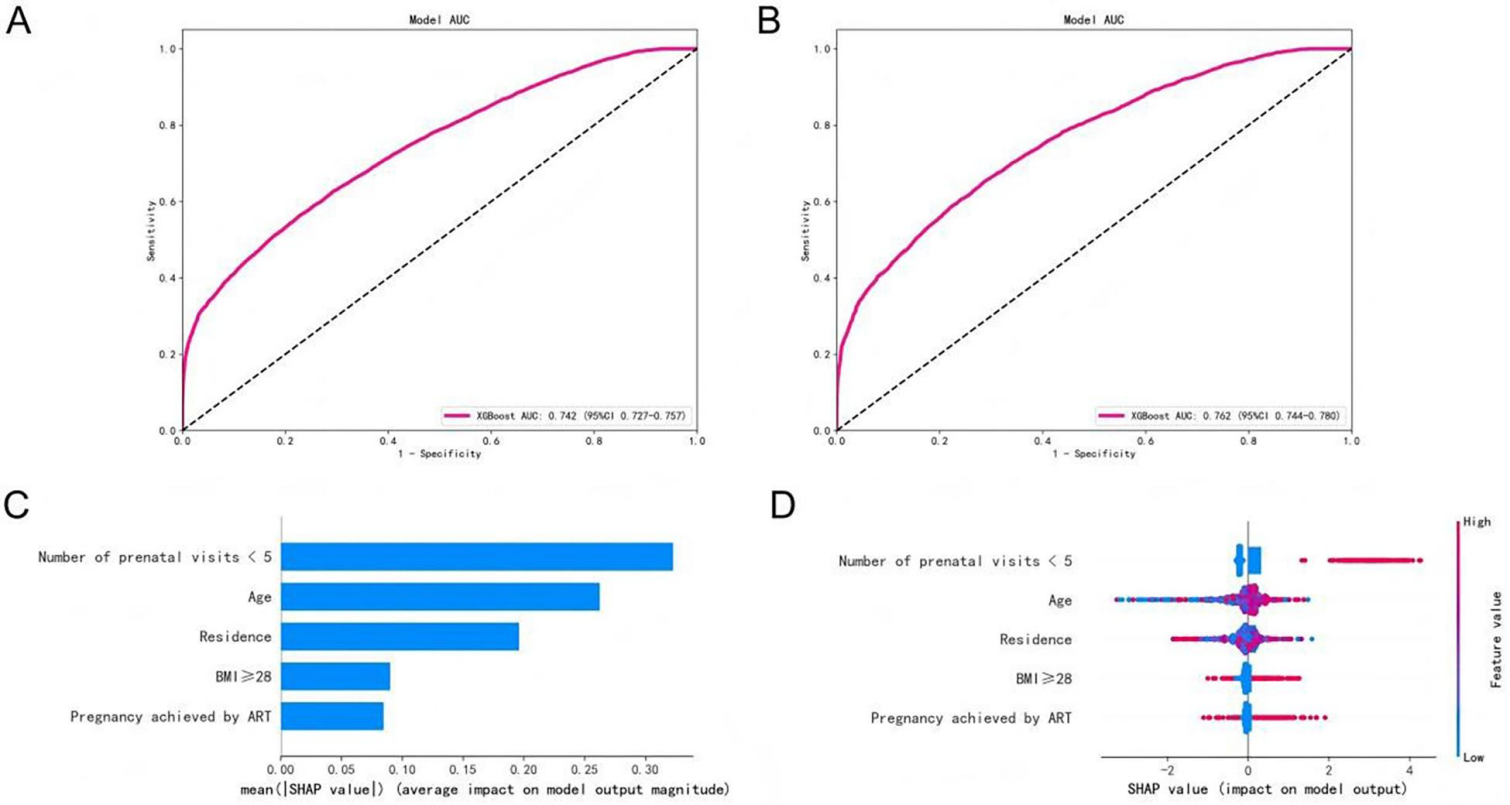
XGBoost model for predicting pregnancy outcomes and SHAP analysis. **(A)** ROC curve for the training set; **(B)** ROC curve for the test set; **(C)** Bar chart of SHAP value rankings; **(D)** SHAP beeswarm plot.

To develop a prediction model for pregnancy outcomes in high-risk pregnancies, the 14,196 high-risk pregnant women were randomly divided into a training set and a test set at a 6:4 ratio. The five variables retained from the multivariate logistic regression analysis (*P* < 0.05) were incorporated into the XGBoost machine learning algorithm to construct the prediction model. The Receiver Operating Characteristic (ROC) curve analysis showed that the model achieved an Area Under the Curve (AUC) of 0.742 (95% CI: 0.727–0.757) in the training set (Figure 5A) and an AUC of 0.762 (95% CI: 0.744–0.780) in the test set (Figure 5B). The SHAP algorithm was employed to interpret the model and determine the importance of each predictor. The top five features, ranked by their mean absolute SHAP value, were: having fewer than 5 prenatal visits, age, place of permanent residence, BMI ≥28 kg/m², pregnancy achieved by ART (Figure 5C). The analysis indicated that a fewer than 5 prenatal visits, higher age, BMI ≥28 kg/m², and pregnancy achieved by ART were associated with an increased risk of adverse pregnancy outcomes (Figure 5D).

## 4 Discussion

This study analyzed data from 19,301 pregnant women in Changde City between July 2022 and July 2023. Among them, 14,196 were assessed as high-risk pregnancies at their initial examination, representing a high-risk pregnancy incidence rate of 73.55%. This rate is substantially higher than the 45.3% reported in a multicenter study from Hebei Province, China (8), and the 38.7% observed in a national survey from India (9). This discrepancy is largely attributable to the comprehensive and sensitive screening criteria mandated by the local five-color risk management policy. This policy proactively classifies a wide array of common, often mild, conditions as “high-risk” to trigger enhanced surveillance, a strategy that differs from the more restrictive definitions used in many other studies. Consequently, our findings reflect the epidemiological landscape under this specific public health framework. Within this high-risk group, 160 individuals (1.13%) experienced adverse maternal outcomes, while 2,041 (14.28%) experienced adverse fetal or neonatal outcomes. Among the adverse maternal outcomes, the most frequent were severe preeclampsia and postpartum hemorrhage. The higher incidence of severe preeclampsia and postpartum hemorrhage among adverse maternal outcomes aligns with global trends (8). Severe preeclampsia can cause organ damage to both the mother and the fetus (10); Postpartum hemorrhage is the leading cause of maternal mortality globally and can lead to significant secondary complications, including adult respiratory distress syndrome, shock, disseminated intravascular coagulation, acute renal failure, loss of fertility, and pituitary necrosis (11). Regarding adverse fetal and neonatal outcomes, preterm birth/low birth weight infants and macrosomia had higher incidence rates. This may be attributed to the various risk factors associated with high-risk pregnancy, which can directly or indirectly affect placental function, the fetal developmental environment, and the progression of pregnancy, ultimately increasing the risk of preterm birth, low birth weight, and macrosomia (12, 13).

Furthermore, this study, utilizing the “Five-Color Risk Management” system, analyzed the most common high-risk factors within each risk level. The top five factors requiring attention in the Yellow (General Risk) category are BMI >25 or <18.5 kg/m², scarred uterus, maternal age >35 or ≤18 years, history of adverse pregnancy/obstetric outcomes, and pregnancy complicated with anemia (Hb 60–110 g/L). For the Orange (Elevated Risk) category, they are: diabetes mellitus, thyroid disorders, or pituitary prolactinoma requiring medication; BMI ≥28 kg/m^2^; history of ≥2 uterine surgeries; maternal age ≥40 years; and scarred uterus (with <18 months interval since the last surgery). Key factors in the Red (High Risk) category include cerebrovascular malformation and surgical history, other severe internal/surgical diseases, active autoimmune/immune system diseases, and generalized epileptic seizures. Regarding the geographic distribution of individuals with high-risk factors across different risk levels, special attention should be given to Wuling District, Hanshou County, Taoyuan County, and Lixian County. Local health departments, medical institutions, and other relevant bodies need to implement targeted measures in areas such as high-risk pregnancy screening and management, as well as the optimization of resource allocation. BMI ≥28 kg/m² was identified as a significant independent risk factor (OR: 1.63). This association may be mediated by the chronic low-grade inflammatory state and insulin resistance characteristic of obesity, which can lead to endothelial dysfunction and increase the risk of preeclampsia, as well as contribute to fetal overgrowth and subsequent delivery complications (14). Fewer than 5 prenatal visits was a strong predictor of adverse outcomes. This likely reflects inadequate surveillance for emerging conditions such as gestational hypertension or diabetes, delaying timely interventions (15, 16). It may also serve as a proxy for other socio-behavioral factors like lower health literacy or barriers to healthcare access. The presence of multiple high-risk factors was associated with a more than two-fold increase in risk. This highlights the synergistic effect of coexisting conditions, where the cumulative physiological burden on the maternal-fetal unit surpasses the threshold for maintaining a healthy pregnancy, leading to a disproportionately higher risk of adverse outcomes.

Currently, artificial intelligence models are seeing increasingly widespread clinical application, enabling the prediction of pregnancy outcomes (17, 18). For instance, Venkatesh et al. considered 55 candidate risk factors available at the time of admission for delivery, using Random Forest and XGBoost to construct a postpartum hemorrhage prediction model (17). Schmidt et al. analyzed 114 features including biomarkers and ultrasound data (umbilical artery pulsatility index, middle cerebral artery pulsatility index, mean uterine artery pulsatility index) to develop an automated machine learning model for predicting adverse outcomes in patients with suspected preeclampsia (18). To further enhance the risk assessment and management of high-risk pregnant women in Changde City, and to implement precise prediction of pregnancy outcomes and risk intervention, this study screened five risk factors influencing pregnancy outcomes using univariate and multivariate logistic regression analyses. Subsequently, a prediction model for pregnancy outcomes in high-risk pregnancies was constructed based on the XGBoost algorithm. The model demonstrated strong predictive performance, with an AUC of 0.742 (95% CI: 0.727–0.757) in the training set and 0.762 (95% CI: 0.744–0.780) in the test set.

The primary contribution of this study lies in its integration of local epidemiological investigation with policy-relevant predictive modeling. Unlike many machine learning studies that rely on standardized, decontextualized datasets, our work is embedded within the operational framework of Hunan Province's five-color management system. This context provides two distinct advantages. First, our descriptive findings on the county-level distribution of specific risk factors (e.g., the high prevalence of scarred uterus in Wuling District or BMI≥28 in Hanshou County) directly inform targeted interventions. Second, the predictive model, once validated, can be seamlessly integrated into the existing electronic health record system (Public Health 3.0) used by local healthcare providers, serving as a real-time clinical decision support tool. Thus, the novelty of this study is not merely methodological, but also lies in its practical applicability within a specific public health ecosystem.

This study also has several limitations: 1) The study duration was relatively short, including only one year of maternal data, we did not perform external validation on an independent dataset or temporal validation on a later cohort. Extending the study period is necessary to expand the sample size and improve temporal representativeness. 2) The follow-up period was brief, lacking long-term tracking of adverse maternal and fetal pregnancy outcomes. Extending the follow-up cycle to conduct long-term longitudinal studies is needed to comprehensively assess the long-term impact of high-risk factors on maternal and infant health. 3) The variables included in building the prediction model were not exhaustive. Future analyses should incorporate more factors, such as environmental elements, economic factors, and specific prenatal examination indicators. 4) This study employed only the XGBoost algorithm to build the machine learning model. Further research could consider using methods like Random Forest or LASSO regression to build and compare models, ultimately selecting the optimal one for clinical application.

In summary, this study provides the first comprehensive, policy-anchored analysis of high-risk pregnancy distribution and its determinants in Changde City. By linking detailed local epidemiological data under the five-color management system with an interpretable machine learning model, we have generated novel, context-specific insights that go beyond generic risk factor identification. The resulting prediction model, once integrated into local health information systems, holds promise as a practical tool for enhancing precision tiered management, thereby contributing to the reduction of adverse maternal and infant outcomes and supporting the goals of the “Healthy China 2030” initiative.

## Data Availability

The raw data supporting the conclusions of this article will be made available by the authors, without undue reservation.
